# Effects of cardiopulmonary bypass on immunoglobulin G antibody titres after SARS-CoV2 vaccination

**DOI:** 10.1093/icvts/ivac123

**Published:** 2022-05-06

**Authors:** Ryosuke Hayashi, Yoshiyuki Takami, Hidetsugu Fujigaki, Kentaro Amano, Yusuke Sakurai, Kiyotoshi Akita, Koji Yamana, Atsuo Maekawa, Kuniaki Saito, Yasushi Takagi

**Affiliations:** 1 Department of Cardiovascular Surgery, Fujita Health University School of Medicine, Toyoake, Japan; 2 Department of Advanced Diagnostic System Development, Fujita Health University Graduate School of Health Sciences, Toyoake, Japan

**Keywords:** Severe acute respiratory syndrome coronavirus 2, Coronavirus disease 2019, Cardiopulmonary bypass, IgG antibody, mRNA vaccine

## Abstract

**OBJECTIVES:**

Patients with cardiovascular disease are vulnerable to severe acute respiratory syndrome coronavirus 2 (SARS-CoV2) infection. Although SARS-CoV2 vaccination may be effective, its impact on surgical patients is not well studied. We investigated the effects of cardiovascular surgery, especially under cardiopulmonary bypass (CPB), on the antibody titres after SARS-CoV2 vaccination.

**METHODS:**

A prospective observational study was designed for patients undergoing surgery between July and November 2021. The immunoglobulin G against the receptor-binding domain was measured and antibody preserved rate (APR) was calculated from perioperative titres comparison.

**RESULTS:**

Enrolled 63 study patients were divided into 39 undergoing surgery with CPB (Group CPB) and 24 without CPB (Group None). Preoperative vaccines were BNT162b2 (Pfizer/BioNTech) (*n* = 58, 92%) and mRNA-1273 (Moderna) (*n* = 5, 8%). While immunoglobulin G against the receptor-binding domain titres did not significantly decrease after surgery in Group None, they decreased significantly in Group CPB from 21.80 [11.15, 37.85] to 11.95 [6.80, 18.18] U/ml (*P* < 0.001) a day after surgery, 11.40 [7.85, 22.65] U/ml (*P* < 0.001) 14 days after surgery and 7.60 [4.80, 17.60] U/ml (*P* < 0.001) a month after surgery. The APRs a day after the surgery were significantly lower in Group CPB (0.46 [0.41, 0.60]) than in Group None (0.80 [0.68, 0.87]) (*P* < 0.001).

**CONCLUSIONS:**

The SARS-CoV2 antibody titres significantly decreased with lower APRs immediately after surgery under CPB. Based on our informative results, careful considerations of vaccination schedule might be required for surgery under CPB.

**Figure:**
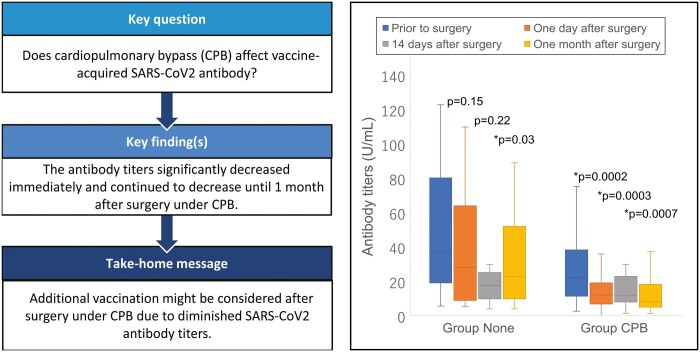

## INTRODUCTION

Since the first outbreak of the severe acute respiratory syndrome coronavirus 2 (SARS-CoV2) in December of 2019, a fear and a threat impacted humanity socially and economically. The SARS-CoV2 is a type of coronavirus which accounts for series of symptoms including fever, myalgia, headache, dyspnoea, cough, sore throat, smell or taste abnormalities, and severe acute respiratory syndrome (SARS) which World Health Organization officially designated as Coronavirus disease 2019 (COVID-19) in February 2020 [1–3]. In a field of cardiovascular surgery, patients suffering from the cardiovascular disease frequently have comorbid medical conditions which are all aggravating factors of COVID-19, including elder, diabetes mellitus, hypertension, chronic respiratory disease and chronic kidney disease [4, 5].

Although pharmaceutical treatments are being developed around the globe, vaccination plays a key role in the prevention of COVID-19. World Health Organization recommends the vaccination for people older than 18 years old, especially for those with vulnerable medical conditions [6]. As of 2021, 3 vaccines are approved in Japan against SARS-CoV2. They are BNT162b2, mRNA-1273 and ChAdOx1-S, developed by Pfizer/BioNTech, Moderna and Oxford/AstraZeneca, respectively. The former 2 are messenger ribonucleic acid (mRNA) vaccines and the latter one is an adenovirus vectored vaccine [7–9].

To determine the efficacy of the SARS-CoV2 vaccine, a serological antibody correlation with vaccine-induced immune response has been reported previously [10]. While the humoral responses after the SARS-CoV2 vaccination in the healthy subjects have been reported, there are no studies related to patients undergoing cardiovascular surgery. Thus, the present study investigated the effects of cardiovascular surgery, especially under cardiopulmonary bypass (CPB), on the patients’ immunoglobulin G (IgG) antibody titres after SARS-CoV2 vaccination.

## PATIENTS AND METHODS

### Ethics statement

This study was approved by Fujita Health University Ethics Committee (HM 21-164). It was also conducted with the informed consent, according to the ethical guidelines for clinical study published by the Ministry of Health and the Declaration of Helsinki.

### Study patients

This prospective observational study in the patients who had already been vaccinated and underwent surgery at Fujita Health University between July 2021 and November 2021.

In this study, patients were divided into 2 groups. The first group included the patients undergone surgery without CPB (Group None), as opposed to the group including the patients undergone surgery with CPB (Group CPB).

### Patient management

In all patients of Group CPB and some patients in Group None, anaesthesia was induced with intravenous midazolam, fentanyl and vecuronium bromide, followed by endotracheal intubation. Anaesthesia was subsequently maintained with sevoflurane, propofol, remifentanil and vecuronium bromide. CPB was performed using a heparin-coated circuit and a membrane oxygenator. In Group CPB, oxygen concentration and ventilation frequency were adjusted to maintain normoxemia (>90 mmHg) and normocapnia (35–45 mmHg). Cardiac arrest was induced by blood-cardioplegia. Pump flow and mean arterial pressure were maintained at 2.5 l/min/m^2^ and above 60 mmHg, respectively, with norepinephrine. Inotropic support with epinephrine was used during CPB weaning.

In some patients of Group None, the surgery was performed with local anaesthesia and sedation with propofol and dexmedetomidine without endotracheal intubation. In both groups, patients were transferred to the intensive care unit (ICU) after the surgery, where they were extubated following institutional criteria.

### Data collection and SARS-CoV2 vaccination

The patient characteristics were collected and analysed from electronic health records, including age at surgery, sex, smoking history and comorbidity (hypertension, hyperlipidaemia, diabetes mellitus, respiratory function, and presence of chronic kidney disease). The surgical procedures performed (type of surgery), a total operation duration, a CPB duration, a cardiac arrest duration, a selective cerebral perfusion duration, total amount of bleeding, an amount of blood transfusion administered, a duration of tracheal intubation, time in ICU and length of hospital stay were recorded.

The SARS-CoV2 vaccination dates and the types of vaccine were interviewed in each patient and were also verified with the vaccination certificates issued by each healthcare institute. Patients with 2 doses of vaccine are enrolled in the study, but those with third booster vaccination are not included. We also checked the history of possible SARS-CoV2 exposure in each patient. The check-ups for SARS-COV2 infection or COVID-19 disease were performed before admission, about 1 week, 2 weeks and 1 month after discharge.

### *Antibody* titre *measurement*

Blood samples for antibody titre measurement were taken at 4 correspondent times, including prior to the surgery, a day after the surgery (7–16 hours after surgery), 2 weeks after surgery and a month after surgery, as visualized in Supplementary Material S1. In addition, when a patient received any blood transfusion, the segment tubes from each pack of the red blood cells were taken for antibody titre measurement.

The IgG against the receptor-binding domain (RBD-IgG) was measured as an antibody titre from each blood samples. A chemiluminescent enzyme immunoassay using an Accuraseed COVID-19 antibody reagent with the Accuraseed automated immunoassay system from FUJIFILM Wako Pure Chemical Corporation was used for the measurement. The details of the method and performance such as limit of quantification, precision, and accuracy were published previously [10].

### Statistical analysis

Categorical data are represented as either a number or a percentage (%) and compared between groups by Fisher's exact test. A level of IgG transition among various times, before and after the surgery, was observed in each group. The differences in change of IgG levels were compared between the 2 groups. Ordinal variables were represented as median with interquartile range. These noncategoric variables were written in the form of median with interquartile range and compared between groups by either a parametric Student’s *t*-test or a nonparametric test, the Mann–Whitney *U*-test. A correlation between 2 variables were analysed using Spearman’s rank correlation coefficient. All statistical analyses were performed with statistical software ‘EZR’ (Easy R) available on website (http://www.jichi.ac.jp/saitama-sct/SaitamaHP.files/statmed.html) [11]. A *P*-value of <0.05 was considered statistically significant.

## RESULTS

**Figure 1:**
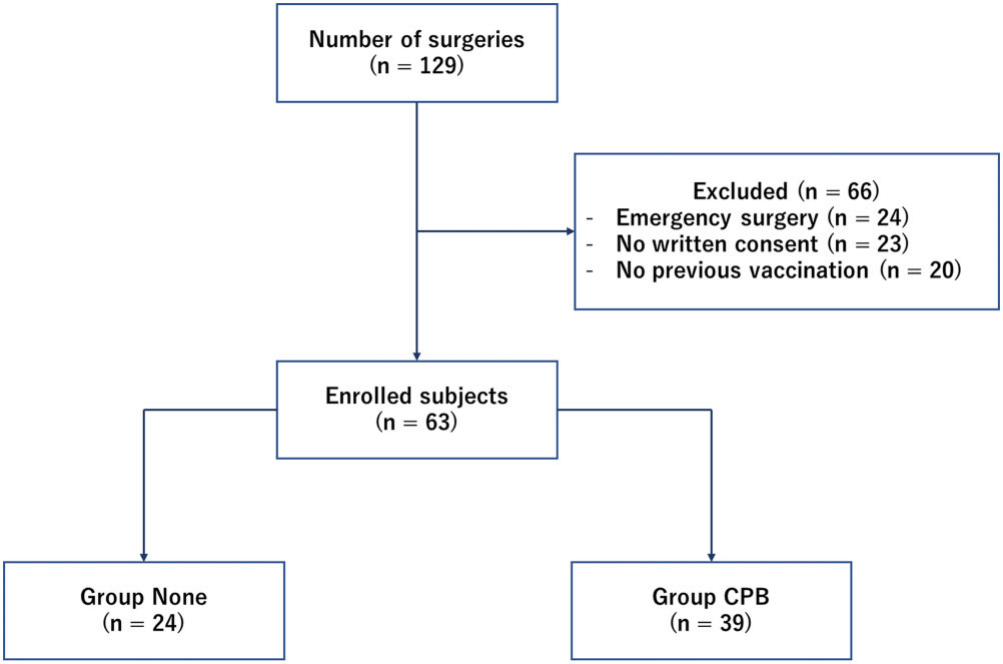
Patients enrolled and excluded in the study.

Between July 2021 and November 2021, there were total of 129 surgical operations performed in our institute. There were 24 unplanned emergency or urgent operations, which were excluded from the research. Twenty-two candidates did not consent to participate in the research, either with their will or lack of ability to do so. Twenty candidates had not vaccinated before the admission. A total of 63 subjects were enrolled to this study (Fig. 1).

The patients in Group None (*n* = 24) underwent endovascular aortic repair (*n* = 17), off-pump coronary artery bypass grafting (*n* = 4), transcatheter aortic valve replacement (*n* = 2) or vascular surgery(*n* = 1). The patients in Group CPB (*n* = 39) underwent on-pump beating coronary artery bypass grafting (*n* = 12), conventional coronary artery bypass grafting (*n* = 4), valve procedure (aortic valve replacement alone; *n* = 2 and mitral valve procedure alone; *n* = 5), aortic procedure (*n* = 6) and combined procedures (*n* = 15), as presented in Table 1. The patient characteristics were not statistically different between the 2 groups, except for smoking history, as also shown in Table 1.

**Table 1:** Patient characteristics and surgical procedures

	Group None (*n* = 24)	Group CPB (*n* = 39)	*P*-Value
Patient characteristics			
Age, years	75.50 [72.75, 80.00]	74.00 [68.50, 77.00]	0.112
Sex, female	7 (29.2)	10 (25.6)	0.777
Hypertension	16 (66.7)	33 (84.6)	0.124
Diabetes mellitus	10 (41.7)	17 (43.6)	1.000
Hyperlipidaemia	16 (66.7)	19 (48.7)	0.198
Chronic lung disease	4(16.6)	7(17.9)	0.614
Smoker	6 (25.0)	22 (56.4)	0.020
Chronic kidney disease	16 (66.7)	24 (61.5)	0.790
Haemodialysis	3 (12.5)	8 (20.5)	0.509
Creatinine, mg/dl	0.98 [0.76, 1.58]	0.97 [0.85, 1.44]	0.596
eGFR, ml/min/1.73 m^2^	51.95 [34.20, 67.83]	56.50 [36.80, 66.15]	0.932
Surgical procedures			
CABG	4	12	
Aortic valve procedure (AVR, TAVI)	2	6	
Mitral valve procedure	–	8	
Aortic procedure (including EVAR and TEVER)	17	6	
CABG + valve procedure	–	4	
Aortic + valve procedure	–	6	
Concomitant atrial fibrillation procedure	–	8	
Concomitant tricuspid procedure	–	2	
Vascular related	1	0	

Data are presented as *n* (%) or interquartile range.

AVR: aortic valve replacement; CABG: coronary artery bypass grafting; CPB: cardiopulmonary bypass; eGFR: estimated glomerular filtration rate; EVAR: endovascular aortic repair; TAVI: transcatheter aortic valve implantation; TEVER: thoracic endovascular aortic repair.

No study patients had past diagnosis of SARS-CoV2 infection based upon our routine check-ups for COVID-19 disease. As for type of SARS-CoV2 vaccines, BNT162b2 (Pfizer/BioNTech) was administered in 58 patients (92%) and mRNA-1273 (Moderna) was administered in 5 patients (8%). The days between the last vaccination and the surgery were significantly longer in Group None (68.50 [50.25, 91.00] days) than in Group CPB (49.00 [24.50, 75.00] days) (*P* = 0.031), as shown in Table 2.

**Table 2:** Measured results

	Group None	Group CPB	*P-*Value
Time between last vaccination to surgery, day	68.50 [50.25, 91.00]	49.00 [24.50, 75.00]	0.031
Vaccination type, BNT162b2	22 (91.7)	36 (92.3)	1.000
Antibody titres, unit/ml			
Prior to surgery	36.85 [18.95, 80.07]	21.80 [11.15, 37.85]	0.050
At 1 day after surgery	28.00 [9.25, 60.40]	11.95 [6.80, 18.18]	0.011
At 14 days after surgery	17.50 [13.60, 20.30]	11.40 [7.85, 22.65]	0.522
At 1 month after surgery	22.50 [11.12, 39.90]	7.60 [4.80, 17.60]	0.002
Operation time, min	172.00 [80.00, 232.50]	344.00 [310.00, 424.50]	<0.001
Cardiopulmonary bypass, min		163.00 [134.00, 211.00]	
Aortic cross-clamp, min (*n* = 25)		128.50 [103.25, 164.00]	
Selective cerebral perfusion, min (*n* = 8)		51.00 [32.00, 108.00]	
Bleeding, ml	200.00 [180.00, 500.00]	1274.00 [879.50, 2026.50]	<0.001
Transfusion			
Red blood cells, unit	4.00 [2.00, 6.00]	11.00 [6.00, 14.00]	0.005
Fresh frozen plasma, unit	10.00 [7.00, 13.00]	12.00 [8.00, 16.00]	0.544
Platelet concentrated, unit		20.00 [20.00, 30.00]	
Antibody contained in transfusion, unit/ml	57.80 [12.32, 123.42]	48.70 [22.30, 77.85]	0.835
Tracheal intubation period, h	0.00 [0.00, 0.75]	14.00 [5.00, 16.50]	<0.001
ICU stay, days	0.00 [0.00, 1.00]	3.00 [2.00, 3.50]	<0.001
Hospital stay, day	8.00 [7.00, 15.50]	23.00 [19.00, 32.00]	<0.001

Data are presented as *n* (%) or interquartile range.

ICU: intensive care unit.

**Figure 2:**
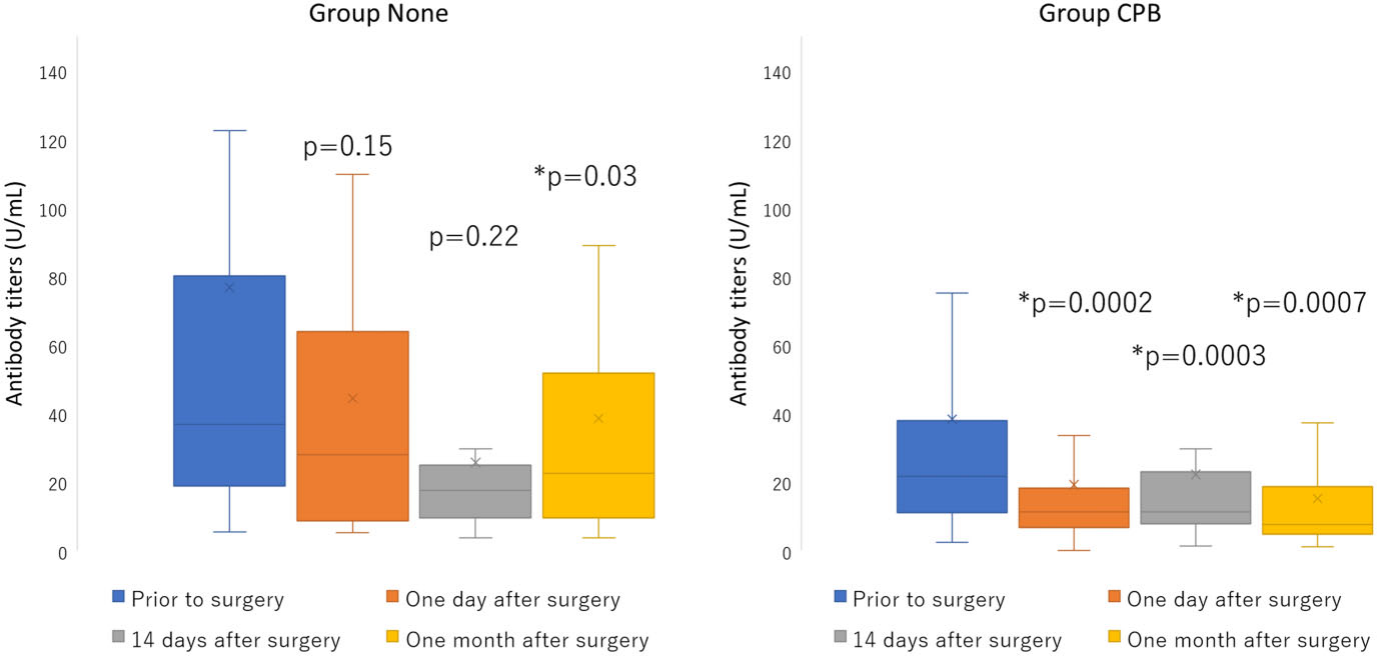
Antibody titres (U/ml) of Group None and Group CPB at various sample timings.

As shown in Table 2 and Fig. 2, the antibody RBD-IgG titres in Group CPB decreased significantly from 21.80 [11.15, 37.85] U/ml before surgery to 11.95 [6.80, 18.18] U/ml (*P *<* *0.001), 11.40 [7.85, 22.65] U/ml (*P *<* *0.001) and 7.60 [4.80, 17.60] U/ml (*P *<* *0.001), a day after surgery, 14 days after surgery and a month after surgery, respectively. In contrast, RBD-IgG titres in Group None did not significantly decrease from 36.85 [18.95, 80.07] U/ml before surgery to 28.00 [9.25, 60.40] U/ml (*P *=* *0.15) a day after surgery and 17.50 [13.60, 20.30] U/ml (*P *=* *0.23) 14 days after surgery. However, the RBD-IgG titres in Group None decreased significantly to 22.50 [11.12, 39.90] U/ml (*P *=* *0.03) a month after surgery, although those were significantly higher than those in Group CPB (*P *=* *0.002). As also shown in Table 2, surgery-related bleeding was significantly more in Group CPB than in Group None; therefore, transfused red blood cells were significantly more in Group CPB. However, the RBD-IgG titres contained in the transfused red blood cells were 57.80 [12.32, 123.42] U/ml in Group None and 48.70 [22.30, 77.85] U/ml in Group CPB (*P *=* *0.835).

**Figure 3:**
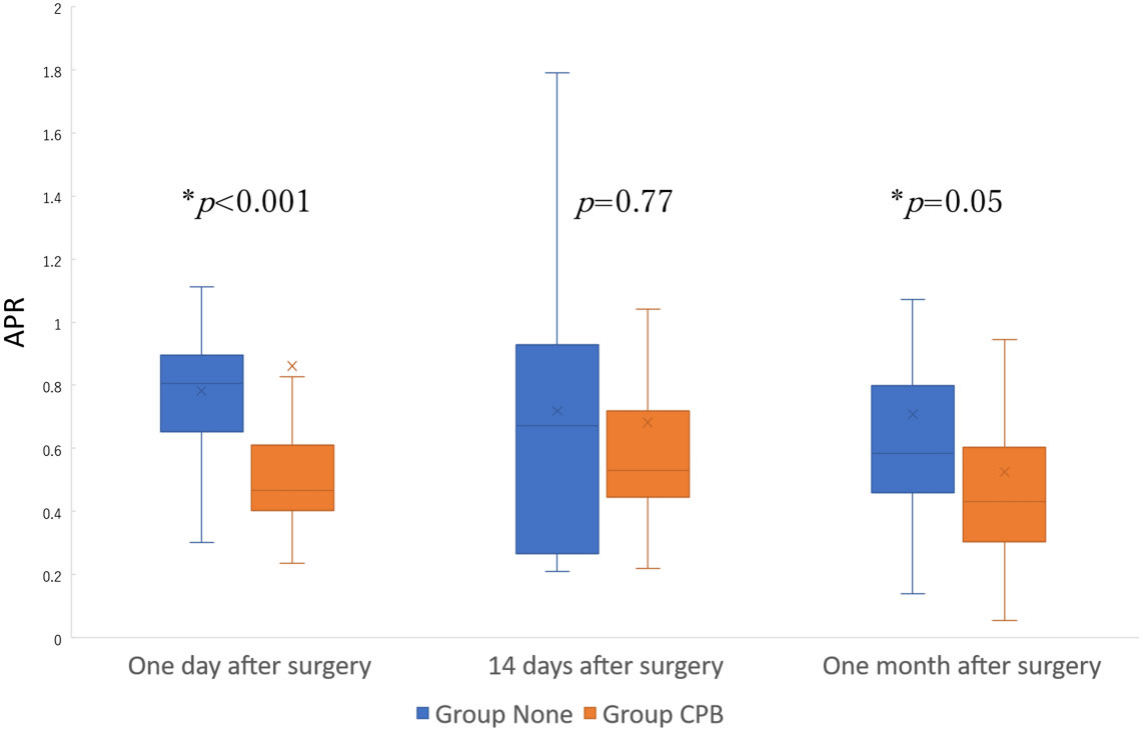
Antibody preservation rate of a day after surgery, 14 days after surgery and a month after surgery.

We labelled antibody preserved rate (APR), calculated from the ratio between the antibody titres before and after the surgery, for the perioperative titres’ comparison. As shown in Fig. 3, the APRs a day after the surgery were significantly lower in Group CPB (0.46 [0.41, 0.60]) than in Group None (0.80 [0.68, 0.87]) (*P *<* *0.001). Also, the APRs a month after the surgery were significantly lower in Group CPB (0.43 [0.31, 0.60]) than in Group None (0.58 [0.48, 0.75]) (*P *=* *0.05). However, the APRs 14 days after the surgery were similar between the 2 groups (*P *=* *0.77): Group None: 0.65 [0.30, 0.92] and Group CPB: 0.52 [0.45, 0.70].

**Figure 4:**
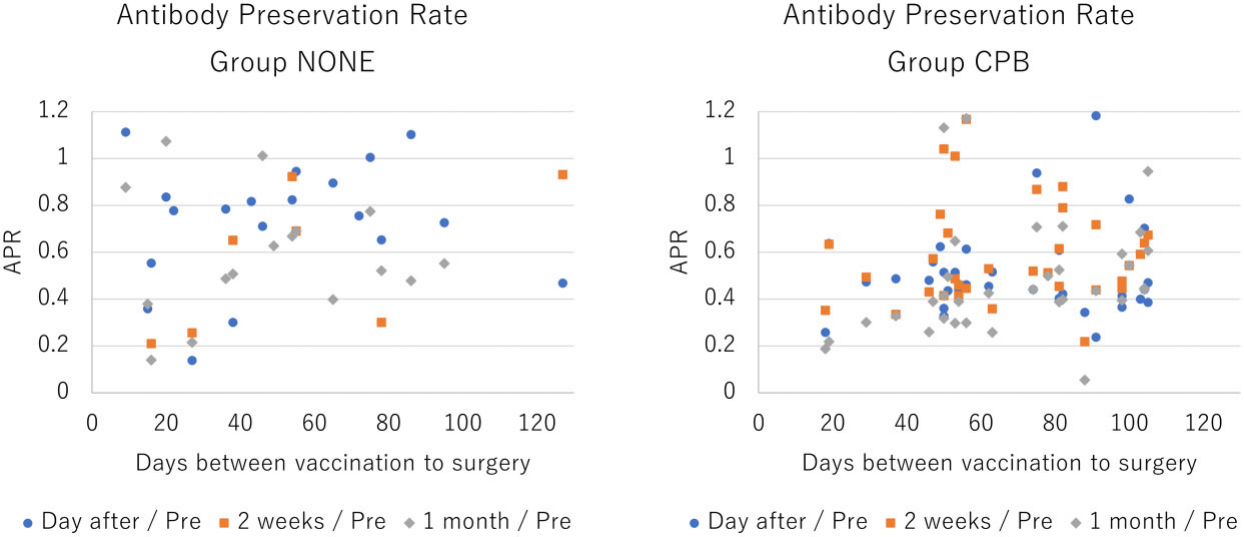
Relationship between antibody preservation rate and interval from vaccination to surgery.

No significant correlations between the vaccination to surgery days and the APRs in both groups a day after (Spearman correlation; Group None: *r* = −0.003, *P* = 0.99 and Group CPB: *r* = −0.122, *P* = 0.48) and 14 days after (Spearman correlation; Group None: *r* = −0.517, *P* = 0.16 and Group CPB: *r* = −0.144, *P* = 0.42) the surgery were observed, as shown in Fig. 4. At a month after the surgery, however, a positive correlation between the interval of vaccination to surgery and the APR was observed in Group CPB (Spearman correlation *r* = 0.488, *P *<* *0.01) but not in Group None (Spearman correlation *r* = 0.131, *P *=* *0.60), as shown in Supplementary Material S2.

There were no relationships between the types of vaccine and the APRs, as shown in Table 3. The APRs a day after surgery were 0.82 [0.41, 0.80] in patients vaccinated with BNT162b2 and 0.59 [0.44, 0.89] in those vaccinated with mRNA-1273 (*P* = 0.76). At 2 weeks after surgery, the APRs were 0.48 [0.00, 0.65] (BNT162b2) and 0.43 [0.25, 0.57] (mRNA-1273) (*P* = 0.85). At a month after the surgery, they were 0.51 [0.27, 0.62] (BNT162b2) and 0.34 [0.22, 0.40] (mRNA-1273), respectively (*P* = 0.43).

**Table 3:** Relationship between vaccination type and antibody preserved ratios

APR	BNT162b2 (*n* = 55)	mRNA-1273 (*n* = 5)	*P-*Value
At 1 day after surgery	0.82 [0.41, 0.80]	0.59 [0.44, 0.89]	0.764
At 14 days after surgery	0.48 [0.00, 0.65]	0.43 [0.25, 0.57]	0.846
At 1 month after surgery	0.51 [0.27, 0.62]	0.34 [0.22, 0.40]	0.432

Data are presented in interquartile range.

APR: antibody preserved rate; CPB: cardiopulmonary bypass.

## DISCUSSION

To our best knowledge, this is the first report investigating the effects of cardiovascular surgery under CPB on the IgG antibody titres after SARS-CoV2 vaccination. The main findings of our study were as follows: (i) There was significant reduction, up to half of the preoperative values, of SARS-CoV2 antibody titres immediately after the surgery in Group CPB. (ii) After a month, antibody titres reduced significantly in both groups. (iii) Although an interval from vaccination to surgery was irrelevant to APR in Group None, positive correlation was observed between the interval and APR at 1 month after the surgery in Group CPB.

Patients queueing cardiovascular surgery are vulnerable to SARS-Cov2 infection not only because of their cardiovascular disease, but along with their background and comorbidities related to their lifestyles. They are recommended for vaccination and priority is high among them [5, 12].

Aside from the novel SARS-CoV2 mRNA vaccine, pre-existed live nor inactive vaccine’s information regarding perioperative vaccination is limited. Even annual influenza vaccination after open heart surgery varies among institutions, lacking consensus [13, 14].

The inflammatory and immune system are known to get altered after any surgery, due to general anaesthesia and its invasive procedure [15, 16]. Furthermore, CPB used in cardiovascular surgery can supplementally disturb those systems [14, 17–19]. Vergales *et al.* [14] reports that the antibody titres did not vary among diphtheria, tetanus, polio1, polio3 or *Haemophiles influenzae* vaccination between before and after the congenital cardiac surgery; however, they had reduced significantly among Bordetella and hepatitis B vaccination. With that in mind, significant reduction of SARS-CoV2 antibody titres in Group CPB but not in Group None in our result is very informative. Although the effect of CPB on the antibody titres was not significantly apparent 14 days after the surgery, there was greater reduction of APR in Group CPB than in Group None after a day and a month, as shown in Figs. 2 and 3.

It is also informative that the antibody titres a month after surgery reduced significantly in both groups. This might reflect the natural wane off of an antibody titre reported in the literature [10, 20, 21] with possible acceleration affected by surgical intervention. A decrease of 75–93% IgG titres after 3 weeks to 3 months after second dose vaccination in healthy subjects is reported previously [10, 20, 21]. Although standard primary SARS-CoV2 vaccination protocol is twice dosage, a third booster or an additional dose is proposed [22, 23]. Our finding might suggest the need of third additional vaccination among post-cardiovascular surgery patients.

A period from vaccination to surgery seemed irrelevant as of APRs in Group None, suggesting that there was no alteration in vaccine acquired immunity after surgery without CPB. However, since a positive correlation between the interval from vaccination to surgery and the APR at a month after the surgery was observed in Group CPB, a careful scheduling of a vaccination may be proposed for surgery under CPB. The lower antibody titres and lower APRs in Group CPB compared to that in Group None a day after and a month after surgery, as shown in Figs. 2 and 3, might have been associated with lower antibody titres before surgery and shorter interval from vaccination to surgery in Group CPB than in Group None. One should bear in mind that the recommended vaccination schedule prior to the surgery is at least 7 days [24, 25], with the context of possible side effects and reactogenicity of the vaccine which approximately takes 10–14 days [7, 8, 25]. No patient had vaccination less than a week before surgery in our study.

### Limitations

There are number of limitations in this research. First, a small number of study sample size influenced the statistical result, making it difficult to analyse the underlying conditions. Also, conduction of single institution research could have added sampling bias. The second limitation is different subject characteristics between 2 comparison groups. Smoking history and vaccination to surgery interval were statistically implicated different in background characteristics. Factors of surgical intervention also weighed more on Group CPB, such as more operation time, more tracheal intubation period, more ICU stay, and more hospital stay, shown in Table 2. The reason behind it is because Group None consisted mostly non-cardiac surgery such as EVAR, TEVAR, and TAVI, contrarily to Group CPB consisted aortic procedure and combined procedures. Third limitation is that study was not a direct comparison between cardiac surgery with and without CPB. Although the difference between Group CPB and Group None in our study may not only be attributed to the usage of CPB, the data of Group CPB in our study is informative in the continuing era of fight against COVID-19 pandemic, armed with new type of mRNA vaccines triggering an immune response. Fourth, the patients enrolled in this study never had third booster dose within the follow-up period. Therefore, our result may be limited since the evidence and data about SARS-CoV2 vaccination are being updated day by day. The fifth limitation is the quantitative measurement of vaccination efficacy. Some advocate RBD-IgG may not necessarily reflect the neutralizing activity against SARS-CoV2 [21]. Although debateable, our previous finding suggests that the monitor of RBD-IgG is plausible and prompt predicative tool to determine the immunogenicity of SARS-CoV2 vaccination [10].

## CONCLUSIONS

A significant reduction of antibody acquired by SARS-CoV2 vaccination was perceived immediately after the surgery with CPB. A positive correlation between the interval from vaccination to surgery and the APR at a month after the surgery was observed in Group CPB, although there was similar tendency of reduction in APR in the patients undergoing with and without CPB. Based upon our informative results, careful considerations of vaccination schedule might be required for the patients undergoing surgery with CPB.

The analysis obtained in this research could support the argument of additional vaccination among perioperative patients. Further study is demanded to provide better preventive medication for perioperative patients. A shared decision-making on additional dose of SARS-CoV2 vaccination among perioperative patients is desired.

## SUPPLEMENTARY MATERIAL

Supplementary material is available at *ICVTS* online.

## Supplementary Material

ivac123_Supplementary_Data

## Data Availability Statement

All relevant data are within the manuscript and its Supporting Information files.

## ABBREVIATIONS

APRs: Antibody preserved rates
COVID-19: Coronavirus disease 2019
CPB: Cardiopulmonary bypass
ICU: Intensive care unit
IgG: Immunoglobulin G
mRNA: Messenger ribonucleic acid
RBD-IgG: IgG against the receptor-binding domain
SARS: Severe acute respiratory syndrome
SARS-CoV2: Severe acute respiratory syndrome coronavirus 2
