# Supplementary figures and images for: Effects of cardiopulmonary bypass on immunoglobulin G antibody titres after SARS-CoV2 vaccination
Source: Interact Cardiovasc Thorac Surg. 2022 May 6;35(3):ivac123. doi: 10.1093/icvts/ivac123 (PMC9383986; doi:10.1093/icvts/ivac123)

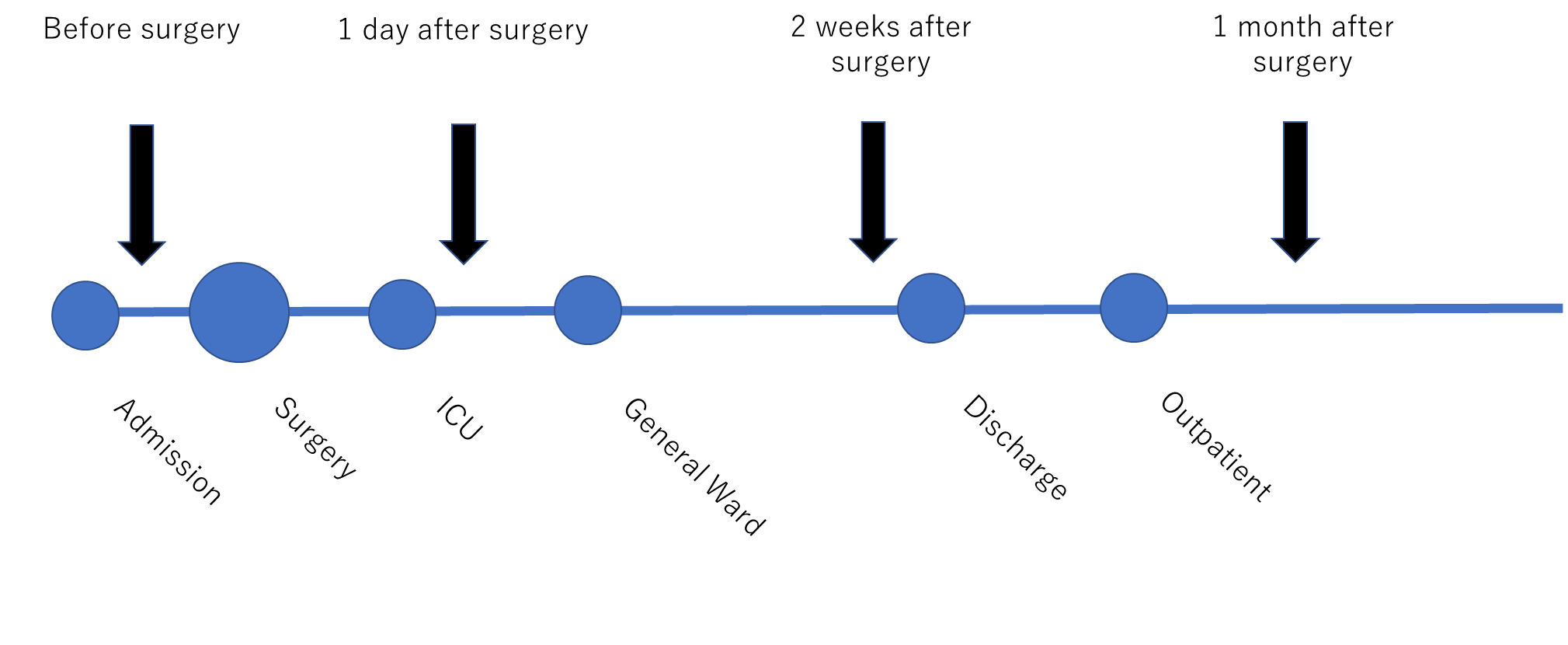

Supplement: ivac123_Supplementary_Data [file ivac123_supplementary_data.zip › ivac123_Supplementary_Data/Supplement 1.tif]

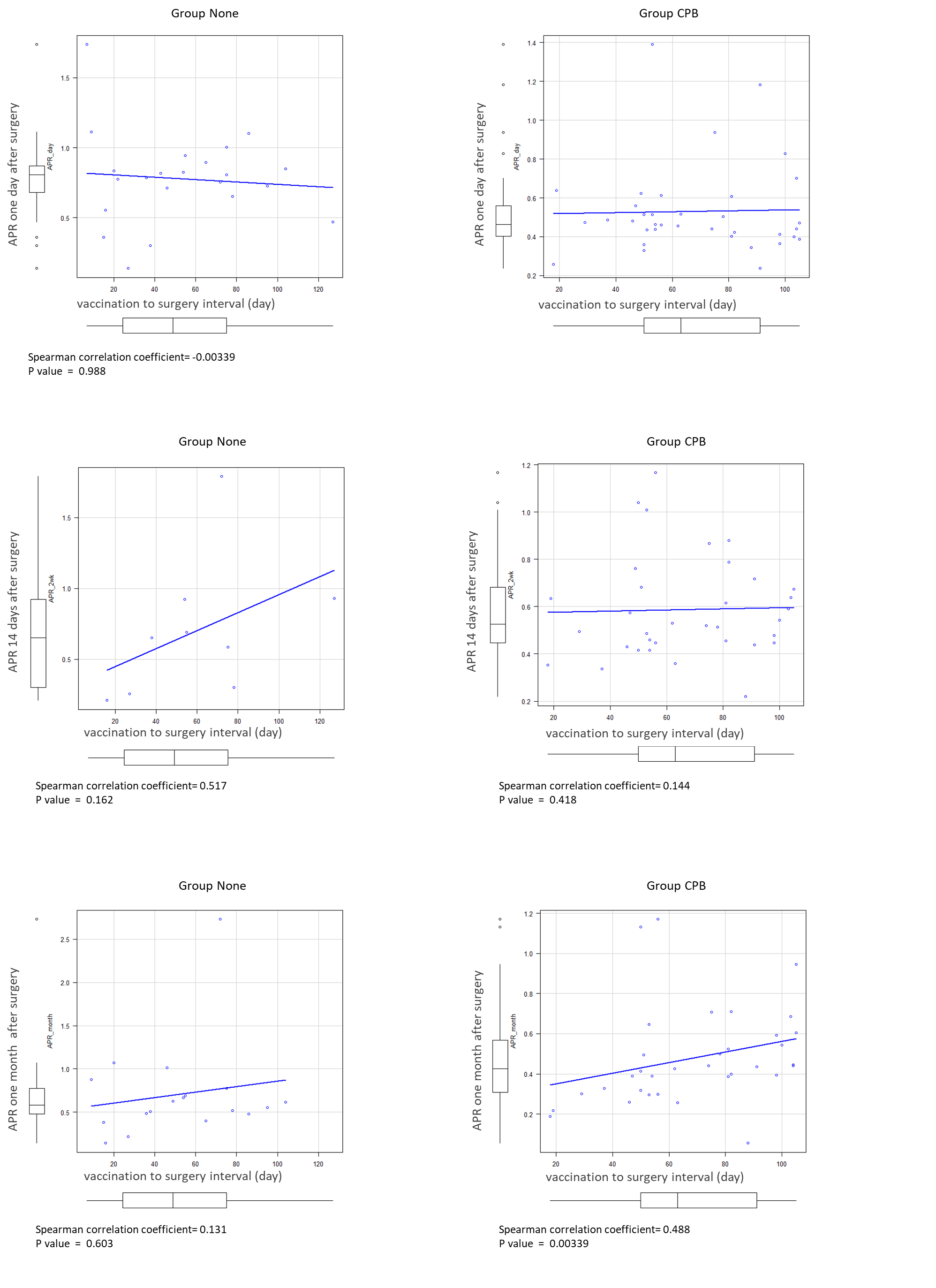

Supplement: ivac123_Supplementary_Data [file ivac123_supplementary_data.zip › ivac123_Supplementary_Data/Supplement 2.tif]
